# Transcriptome-Wide Binding Sites for Components of the *Saccharomyces cerevisiae* Non-Poly(A) Termination Pathway: Nrd1, Nab3, and Sen1

**DOI:** 10.1371/journal.pgen.1002329

**Published:** 2011-10-20

**Authors:** Tyler J. Creamer, Miranda M. Darby, Nuttara Jamonnak, Paul Schaughency, Haiping Hao, Sarah J. Wheelan, Jeffry L. Corden

**Affiliations:** 1 Department of Molecular Biology and Genetics, The Johns Hopkins University School of Medicine, Baltimore, Maryland, United States of America; 2 High Throughput Biology Center, The Johns Hopkins University School of Medicine, Baltimore, Maryland, United States of America; 3 Department of Oncology, The Johns Hopkins University School of Medicine, Baltimore, Maryland, United States of America; 4 Department of Biostatistics, Bloomberg School of Public Health, The Johns Hopkins University, Baltimore, Maryland, United States of America; University of Oxford, United Kingdom

## Abstract

RNA polymerase II synthesizes a diverse set of transcripts including both protein-coding and non-coding RNAs. One major difference between these two classes of transcripts is the mechanism of termination. Messenger RNA transcripts terminate downstream of the coding region in a process that is coupled to cleavage and polyadenylation reactions. Non-coding transcripts like *Saccharomyces cerevisiae* snoRNAs terminate in a process that requires the RNA–binding proteins Nrd1, Nab3, and Sen1. We report here the transcriptome-wide distribution of these termination factors. These data sets derived from in vivo protein–RNA cross-linking provide high-resolution definition of non-poly(A) terminators, identify novel genes regulated by attenuation of nascent transcripts close to the promoter, and demonstrate the widespread occurrence of Nrd1-bound 3′ antisense transcripts on genes that are poorly expressed. In addition, we show that Sen1 does not cross-link efficiently to many expected non-coding RNAs but does cross-link to the 3′ end of most pre–mRNA transcripts, suggesting an extensive role in mRNA 3′ end formation and/or termination.

## Author Summary

Transcription in eukaryotes is widespread including both protein-coding transcripts and an increasing number of non-coding RNAs. Here we present the results of transcriptome-wide mapping of a set of yeast RNA–binding proteins that control expression of some protein-coding genes and a number of novel non-coding RNAs. The yeast Nrd1-Nab3-Sen1 pathway is required for termination and exosome-mediated processing of non-coding RNA polymerase II transcripts. Our data show that these components bind unexpected targets including a large number of antisense transcripts originating from the 3′ end of genes that are poorly expressed in the sense direction. We also show that Sen1 helicase, involved in termination of non-coding RNAs, is also present at the 3′ end of mRNAs, suggesting a more fundamental role in transcription termination. Mis-regulation of transcription is the underlying cause of many disease states. For example, mutation of the human Sen1 gene, senataxin, causes a range of neurodegenerative disorders. Understanding the roles of yeast RNA–binding proteins in controlling termination of coding and non-coding RNAs will be useful in deciphering the mechanism of these proteins in human cells.

## Introduction

Early in each transcription cycle RNA polymerase II (Pol II) can follow one of two paths; terminate early through the Nrd1-Nab3-Sen1 pathway or continue on to form longer, potentially coding transcripts [1], [2]. Yeast Nrd1, Nab3 and Sen1 are part of a complex [3] that interacts both with the phosphorylated Pol II C-terminal domain (CTD) [4]–[6], and with specific sequences in the nascent transcript [7], [8]. If this set of protein-protein and protein-RNA contacts is sufficient, the Nrd1-Nab3-Sen1 complex directs Pol II termination and couples this to processing of the nascent transcript by TRAMP and the nuclear exosome [9], [10]. In addition to directing termination of snoRNAs and cryptic unstable transcripts (CUTs) the Nrd1-Nab3-Sen1 pathway directs premature termination of several pre-mRNA transcripts including *NRD1*, *IMD2*, *URA2*, *URA8*, and *ADE12* [11]–[15]. The mechanism by which the Nrd1-Nab3-Sen1 complex leads to Pol II termination is unknown but involves recognition of specific terminator sequences by Nrd1 and Nab3 [7], [8], [16] and the putative helicase activity of Sen1 [16], [17].

In this study we have used a recently developed in vivo cross-linking approach [18] to derive high-resolution transcriptome-wide maps of binding sites for Nrd1, Nab3, Sen1, and the Pol II subunit Rpb2. This approach yields a more precise picture of known Nrd1 and Nab3 binding sites on snoRNA, CUT and mRNA targets and reveals a set of previously unknown Nrd1 binding sites both on the 5′ ends of mRNAs and on 3′ antisense transcripts. Surprisingly, Sen1 does not cross-link at many of these Nrd1-Nab3 binding sites. Instead, we observe Sen1 cross-linking on mRNA transcripts, particularly at the 3′ end suggesting a potential role for Sen1 in mRNA 3′ end formation through the cleavage and polyadenylation pathway.

## Results

The Photoactivatable-Ribonucleoside-Enhanced Crosslinking and Immunoprecipitation (PAR-CLIP) technique [18] was adapted to yeast (Materials and Methods). Briefly, cells are grown in the presence of 4-thiouracil which is incorporated into RNA. UV irradiation at 365 nm results in high-efficiency covalent cross-linking of RNA to protein [18]. We then purify the tagged protein under denaturing conditions [19], prepare cDNA libraries to the covalently attached RNAs and sequence the libraries. As previously reported, reverse transcription of the cross-linked 4-thiouracil leads to a characteristic T to C transition in the cDNA sequence [18]. Among all single-base changes observed in the sequences we derived from our initial Nrd1 and Nab3 cross-linking experiments approximately 90% were T to C transitions; about 10-fold greater than the number expected for random errors. Data analysis was limited to sequences containing a single base change from the reference sequence thus pinpointing the site of cross-linking in these sequences.

In all, we report sequence data for five libraries (Table S1) including data for Nrd1, Nab3, Sen1 and the RNA Pol II subunit Rpb2. Similar datasets have recently been reported by the Tollervey lab [20] who crosslinked Nrd1, Nab3 and Trf4 using an alternative UV cross-linking procedure (CRAC) [21]. The Nrd1 and Nab3 cross-linking datasets obtained by PAR-CLIP and CRAC are very similar. In both cases the majority of cross-links occur on Pol II transcripts with snoRNA transcripts the most abundant. Pol III transcripts are also cross-linked to Nrd1 and Nab3 and Wlotzka et al. provide evidence for a role for Nrd1 in processing pre-tRNAs [20]. A minor amount of Nrd1, Nab3 and Sen1 cross-links to ribosomal RNA and we have not pursued this further. Our cross-linking of Nrd1, Nab3, Sen1 and Rpb2 to snoRNA, snRNA, and tRNA will be discussed in another publication [22].

To validate the PAR-CLIP procedure in yeast we identified Nrd1 and Nab3 binding sites downstream of the *SNR13* gene (Figure S1) with a peak of cross-linking within a region containing termination elements identified through genetic analysis [7], [8]. In addition, Figure S1 shows multiple Nrd1 and Nab3 binding sites in the upstream region of the Nrd1 gene, an observation consistent with our previous work showing that multiple mutations were required to inactivate autoregulation of Nrd1 expression [11]. While we cannot say with certainty whether the extent of cross-linking is exhaustive, we observe cross-linking at all previously observed Nrd1 and Nab3 binding sites including those on snoRNAs, snRNAs, attenuated mRNA transcripts and CUTs.

### RNA binding is not required for Nrd1 chromatin association

**Figure 1:**
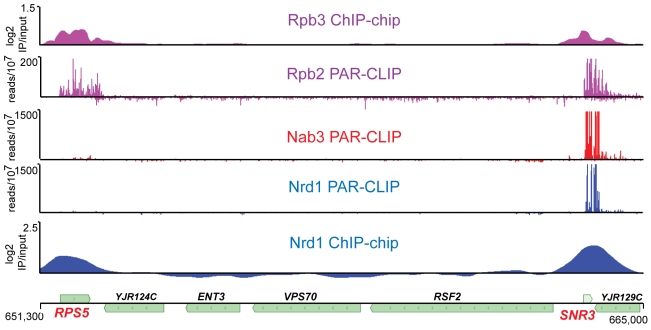
Transcriptome and chromatin mapping of Nrd1, Nab3, and Pol II on a segment of chromosome X. The top two panels depict the position of Pol II. The bottom two panels show the position of Nrd1 and the middle panel depicts Nab3. The top and bottom panels represent chromatin immunoprecipitation data for Rpb3 [63] and Nrd1 expressed as log2 IP/input, while the middle three panels represent RNA cross-linked to Rpb2, Nrd1 and Nab3 expressed as reads per 10^7^ reads in the respective data sets. The scale for Nrd1 and Nab3 PAR-CLIP has been set to 500×10^7^ to emphasize the lack of PAR-CLIP reads at *RPS5*. The peak of reads (×10^7^) for Nrd1 and Nab3 on snR3 transcripts is 3,000 and 7,000, respectively. The coordinates and positions of genes on chromosome X are shown at the bottom.

Previous studies have shown that Nrd1 and Nab3 are present on chromatin and localized to genes that are regulated by Nrd1 and Nab3 [4], [6], [9], [11], [23]. Because the Nrd1 complex interacts both with the nascent transcript and with Pol II, it is not clear which interaction is primarily responsible for chromatin binding. We have analyzed Nrd1 and Nab3 interactions with both chromatin and RNA. Figure 1 shows a comparison of ChIP-chip of Nrd1 compared to Nrd1 and Nab3 PAR-CLIP. Two tracks displaying Pol II subunits Rpb2 and Rpb3 are shown for reference, as they are both part of the catalytic core and thus are expected to co-localize. All five data sets were obtained from cells growing in log phase. Nrd1 and Pol II co-localize by ChIP at both the *RPS5* and *SNR3* genes (Figure 1). Nab3 ChIPs similarly to Nrd1 (not shown). In contrast, Nrd1 and Nab3 cross-link to RNA in a subset of the major Nrd1 ChIP peaks. For example, in Figure 1 we observe efficient cross-linking of Nrd1 and Nab3 to *SNR3* transcripts but not to *RPS5* transcripts. Genomewide, the Nab3 RNA cross-linking pattern is nearly identical to Nrd1 (Wilcoxon rank sum p<10^−8^). Similar specificity of Nrd1 RNA cross-linking is seen at other highly expressed genes. Among the top 100 Nrd1 ChIP peaks determined using CisGenome [24] are ten other ribosomal protein genes, none of which are among the top 100 Nrd1 PAR-CLIP peaks (not shown). We conclude that Nrd1, Nab3, and presumably Sen1 are able to enter the early Pol II elongation complex independent of RNA binding and monitor the nascent RNA for appropriate binding sites.

### Nrd1 and Nab3 binding sites

**Figure 2:**
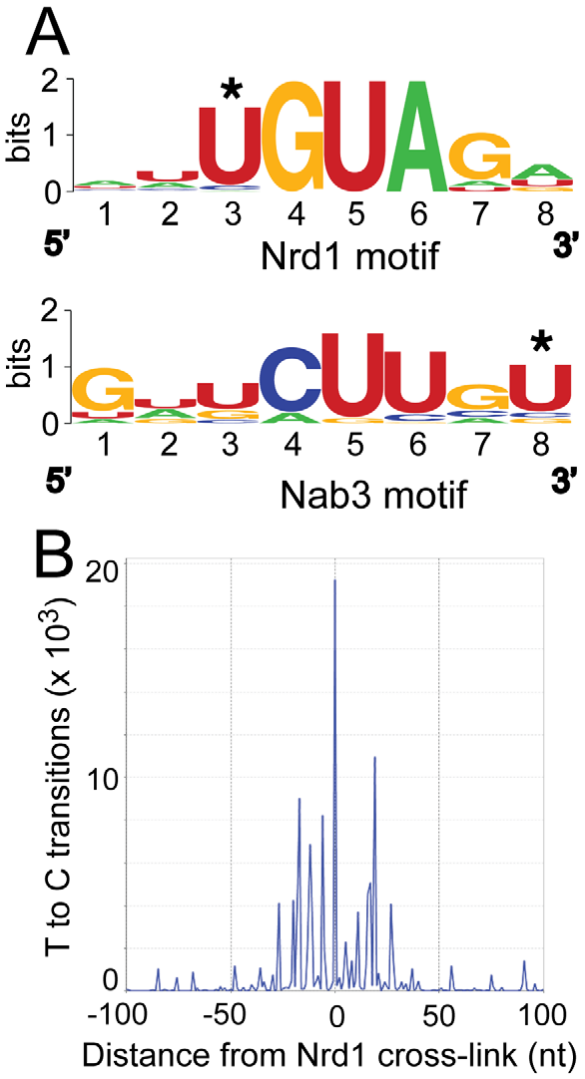
Conserved Nrd1 and Nab3 binding motifs. (A) Sequence Logos of Nrd1 and Nab3 cross-linking sites generated from MEME analysis of the top 277 cross-linked sites for Nab3 and the top 271 cross-linked sites for Nrd1. MEME conditions were set to discover zero or one motif per sequence with a minimum number of sites set to 100 and the width to eight nucleotides. (B) Histogram showing the distribution of T to C transitions plotted with respect to the top 50 Nrd1 cross-linking sites anchored to the zero position on the X axis.

Figure 2A shows the logos [25] of motifs in a representative MEME [26] trial using sequences of 40 nt centered on the most prominent RNA cross-link (T to C) sites. Nrd1 cross-links reveal a consensus sequence UGUAG with an E-value of 1.8e^−25^ where the underlined U is the most frequently cross-linked residue. The top-scoring motif in the Nab3 pool is a consensus sequence GNUCUUGU . The E-value under the same conditions as the Nrd1 analysis is much higher (6.7e^3^) indicating that this motif is not nearly as constrained. These motifs are nearly identical to the GUA[A/G] and UCUU sequences that we previously identified using genetic and biochemical approaches [7], [8] and contain many of the over-represented 4 mer sequences in sequences that cross-link to Nrd1 and Nab3 by the CRAC protocol with 254 nm UV irradiation [20]. Our Nab3 motif is very similar to the UUCUUGUW motif identified by microarray analysis of RNA co-purified with Nrd1 in the absence of cross-linking and under non-denaturing conditions [27]. No conserved motifs were observed for Sen1 and Rpb2, consistent with their presumed roles as enzymes that interact non-specifically with RNA.

About 50% of the Nrd1 and Nab3 cross-linked regions overlap. This is not surprising given that Nrd1 and Nab3 are known to dimerize in vivo and in vitro [7], [28]. The second most significant motif, as determined in MEME, in the Nrd1 set corresponded to the top rated motif in the Nab3 analysis and vice versa, again suggesting that Nrd1 and Nab3 binding sites are located close to each other.

In Figure 2B we show that Nrd1 cross-linking sites are clustered. The 50 Nrd1 cross-linked sites with the most reads (as determined by MochiView [29]) were used as an anchor in this plot. The number of cross-links observed as a function of the distance from the top 50 cross-linked sites is increased in a window approximately 30 nt upstream and downstream of the central Nrd1 cross-link. No similar clustering of Nab3 cross-linking sites was observed indicating that most Nab3 binding regions do not contain multiple motifs. Together, these results suggest that these strong Nrd1 binding sites have been selected to bind multiple Nrd1 proteins, a result consistent with our *in vitro* binding experiments suggesting that the Nrd1-Nab3 complex binds cooperatively to RNA targets [7].

### Nrd1, Nab3, and Sen1 bind to snoRNA transcripts

**Figure 3:**
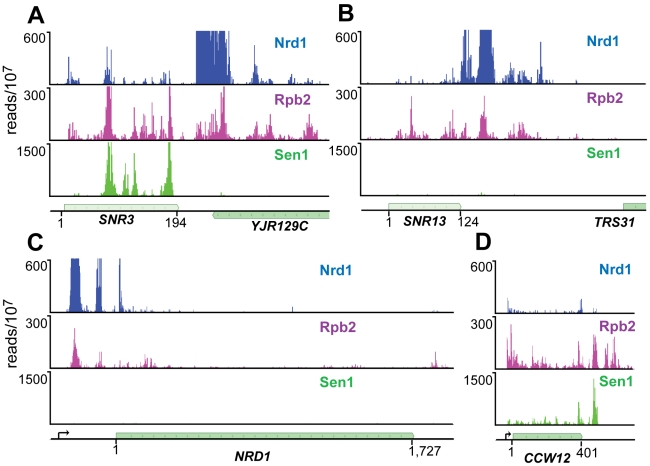
PAR-CLIP plots for Nrd1, Rpb2, and Sen1. (A) and (B) Binding to snoRNA genes *SNR3* and *SNR13*. (C) and (D) Binding to protein coding genes *NRD1* and *CCW12*. Pre-mRNA transcription start-sites are indicated by arrows on the gene map. The Nrd1 scale is set to 600 reads/10^7^ to emphasize minor peaks. Full-scale peaks for Nrd1 on *SNR3* and *SNR13* are 5,000 reads/10^7^ and for the *NRD1* gene is 2,500 reads/10^7^.

For both Nrd1 and Nab3 data sets binding sites on snoRNA transcripts were among the most extensively cross-linked (Tables S2 and S3). At most snoRNAs we observed a peak of Nrd1 binding downstream of the mature 3′ end of the RNA consistent with the previously demonstrated role in termination [16]. Figure 3A and 3B shows the distribution of cross-linked sequences downstream of *SNR3* and *SNR13*. Nrd1 binds predominantly downstream of the mature RNA, while Rpb2 cross-links across the transcript. Surprisingly, Sen1 cross-links efficiently to some snoRNA transcripts like snR3, but not to others, like snR13. This is an unexpected result considering previous experiments showing that at both of these snoRNA genes Pol II reads through the terminator in a *sen1* mutant at the non-permissive temperature [16] and both genes have Sen1 present on chromatin as determined by ChIP [30], [31].

### Sen1 binds poorly to attenuated mRNA transcripts

We have previously shown that Nrd1 regulates its own expression by binding to sites in the 5′ end of its mRNA and directing premature termination. This attenuation mechanism requires the function of Nrd1, Nab3 and Sen1 [16]. The Nrd1-Nab3-Sen1 pathway is also required for the formation of attenuated transcripts from the *IMD2* promoter [12]. We were therefore surprised that Sen1 does not crosslink as efficiently to *NRD1* or *IMD2* mRNA as it does to other mRNAs, for example *CCW12* (Figure 3C and 3D and Figure S2).

### Nrd1 binds preferentially to unstable intergenic ncRNAs

Of the top 300 Nrd1 and Nab3 peaks, 93 and 54, respectively, overlap with CUTs (out of 925 annotated CUTS [32], [33]). By contrast, only 23 (Nrd1) and 8 (Nab3) peaks overlap with SUTs (stable unannotated transcripts, out of 847 annotated SUTs [32], [33]). This preference for binding unstable transcripts is consistent with Nrd1-Nab3 binding being a key feature that distinguishes between CUTs and SUTs [20]. In Figure S3 we show that Nrd1 binds to several known CUTs. The Nrd1 cross-linking sites on CUT transcripts tend to be located toward the 5′ end, a position in which Nrd1 and Nab3 may direct the observed range of termination sites downstream of these binding sites [9], [10], [34]. The observation that some CUTs are not bound by Nrd1 is somewhat surprising. In Figure S3 we observe that some CUTs and SUTs are not expressed under our experimental conditions as shown by the lack of cross-linking to Rpb2.

### Nrd1 and Sen1 are differentially distributed on protein-coding genes

**Figure 4:**
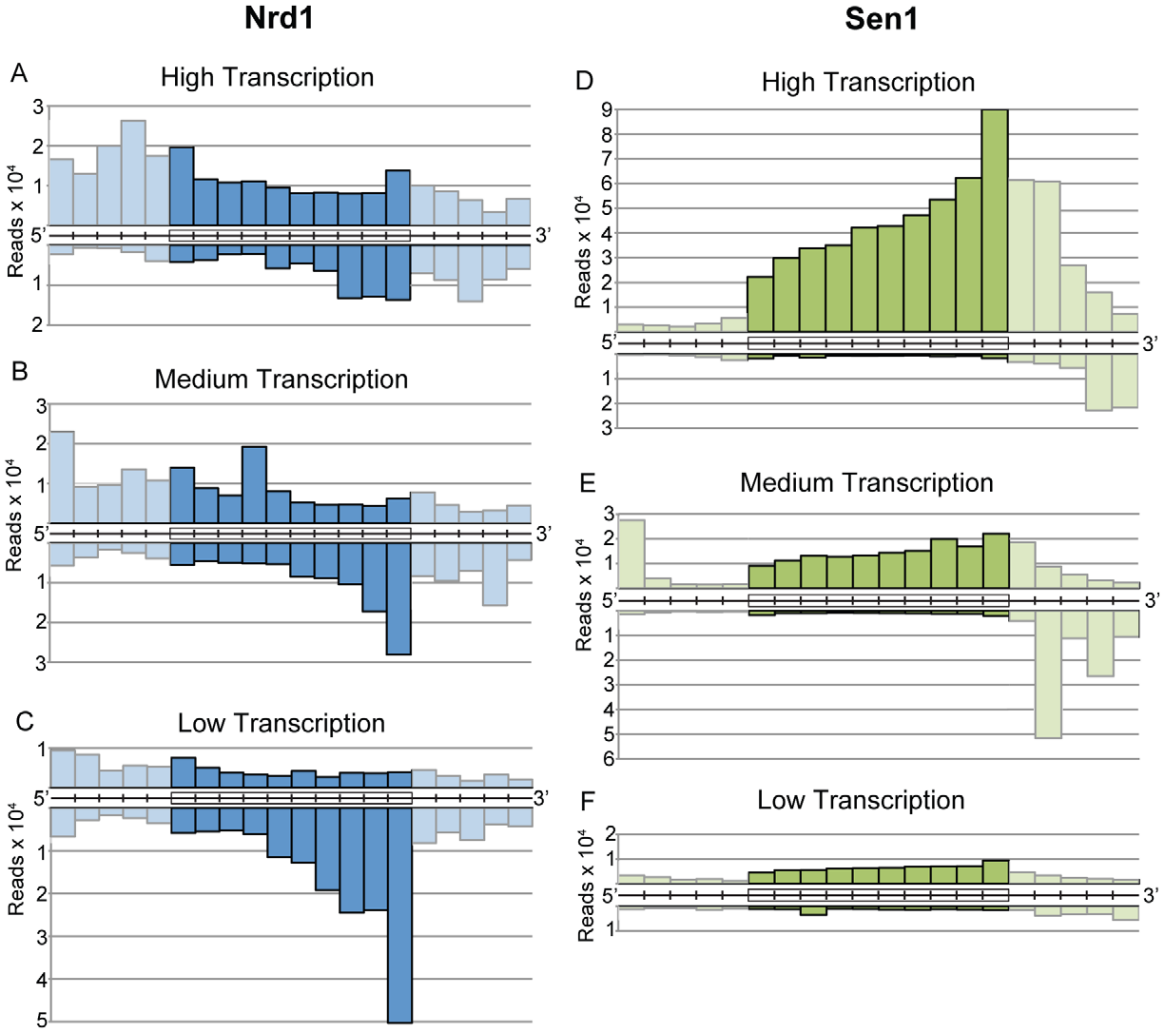
Gene-averaged positive and negative strand Nrd1 and Sen1 cross-linked reads on genes ranked by expression level. (A–C) Nrd1. (D–F) Sen1. High transcription and low transcription are the top and bottom quartiles as determined by RNA sequencing [39]. Medium transcription represents the middle half of yeast genes ranked by expression level. Cross-linked RNA reads from the positive strand (above) and negative strand (below) were sorted into ten fractionally equal coding-region segments (indicated by boxes) and 50 bp segments for the 5′ and 3′ UTRs. Lighter shading is used to indicate the difference between scaled reads derived from coding regions and unscaled reads from the UTRs.

Nrd1, Nab3, and Sen1 also bind to RNA sequences derived from protein-coding genes. Cross-linked sequence reads were sorted into ten bins covering each coding region in order to display genes of different length on the same scale. Figure 4A–4C shows the distribution of Nrd1 plus-strand and minus-strand reads on genes ranked by expression level. Figure 4D–4F shows a similar distribution of Sen1 reads. Highly expressed genes have a peak of Nrd1 sense-strand reads derived from the 5′ end. Genes with the lowest level of expression show a peak of Nrd1 cross-linked antisense reads at the 3′ end. For Sen1 we observe the largest number of reads on the 3′ end of the most abundantly transcribed genes.

### Nrd1 binding to the 5′ end of protein-coding transcripts

Nrd1 has been implicated in several regulatory mechanisms involving binding to sequences in the 5′ end of transcripts. Autoregulation of Nrd1 expression by attenuation is one example. Another form of regulation involving Nrd1 and Nab3 binding near the 5′ end of transcripts is alternative transcription start site (TSS) selection on genes involved in nucleotide biosynthesis. *IMD2*, *URA2*, *URA8* and *ADE12* have been shown to use alternative transcription start sites (TSSs) in response to nucleotide availability. For *IMD2* and *URA2* the upstream starts used in the presence of sufficient NTP result in the elongation complex passing through a Nrd1-Nab3 terminator thus reducing transcription of mRNA [12], [13], [15]. We observe peaks of Nrd1 binding on all of these transcripts (not shown). In addition, we see similar binding on other genes involved in nucleotide metabolism including *HPT1*, *GUA1* and *ADE17* (Figure S4). For each of these genes multiple TSSs have been reported and are located upstream and downstream of the Nrd1-Nab3 binding region, consistent with regulation by TSS selection.

**Figure 5:**
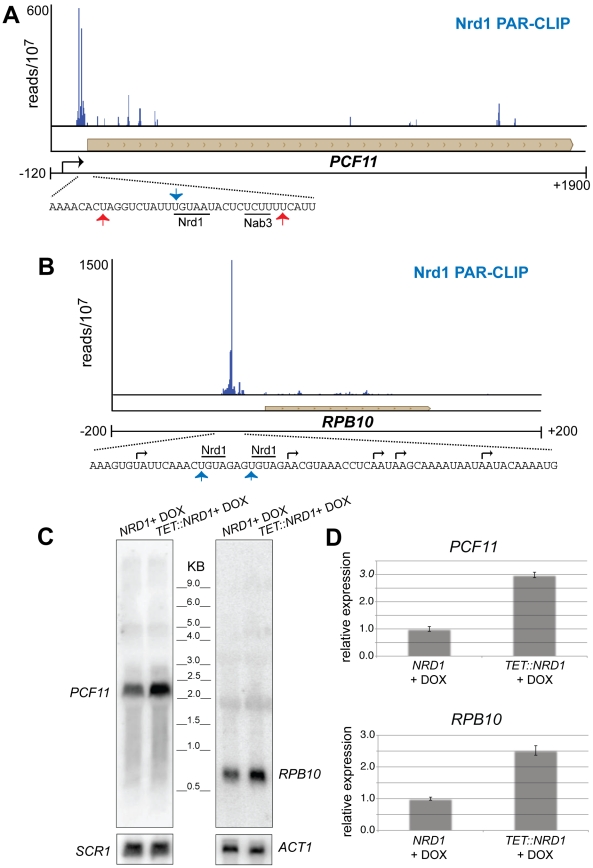
Nrd1 regulation by binding in the 5′ UTR. (A and B) Nrd1 cross-linking to the 5′ UTR of *PCF11* and *RPB10*. The sequence below each plot shows the Nrd1 (blue arrow) and Nab3 (red arrows) cross-linking sites. Transcription start sites are indicated by black arrows. (C) Northern Blot for *PCF11* and *RPB10* transcripts derived from wild-type cells or cells in which Nrd1 expression is regulated by a Tet promoter [62]. Cells were grown in the presence of doxycycline. (D) Quantitative RT-PCR of *RPB10* and *PCF11* RNA derived from wild-type cells or cells in which Nrd1 expression is regulated by a Tet promoter [64]. Cells were grown in the presence of doxycycline. The relative abundance of each target transcript is normalized to the abundance of *ACT1* and 18S rRNA in the same sample.

Figure 5 shows two additional genes, *PCF11* and *RPB10* that have 5′ Nrd1 binding peaks. In each case the peak of binding is downstream of at least one mRNA 5′ end suggesting that the gene may be regulated by premature termination. Increased levels of RNA from cells in which Nrd1 levels were depleted confirm that *PCF11* and *RPB10* are negatively regulated by Nrd1 (Figure 5C and 5D). In the case of *PCF11* this regulation is particularly interesting because *PCF11* encodes a protein with a similar termination function to Nrd1. While Pcf11 has primarily been associated with termination of mRNAs it also plays a role in termination of non-poly(A) transcripts 30,35. Nrd1 and Pcf11 have been proposed to compete for recruitment to the transcription complex [36], [37] and the ability of Nrd1 to regulate Pcf11 expression may play a role in balancing this competition. For example, conditions that reduce Nrd1 protein levels would be expected to lead to compensatory increases in both *NRD1* and *PCF11* expression. Nrd1 binding also regulates expression of *RPB10*, a gene encoding an RNA polymerase subunit that is common to all three nuclear RNA polymerases. This observation suggests a possible role for the Nrd1 pathway in regulating expression of the transcription machinery.

### Nrd1 binds to antisense transcripts at the 3′ end of coding regions

**Figure 6:**
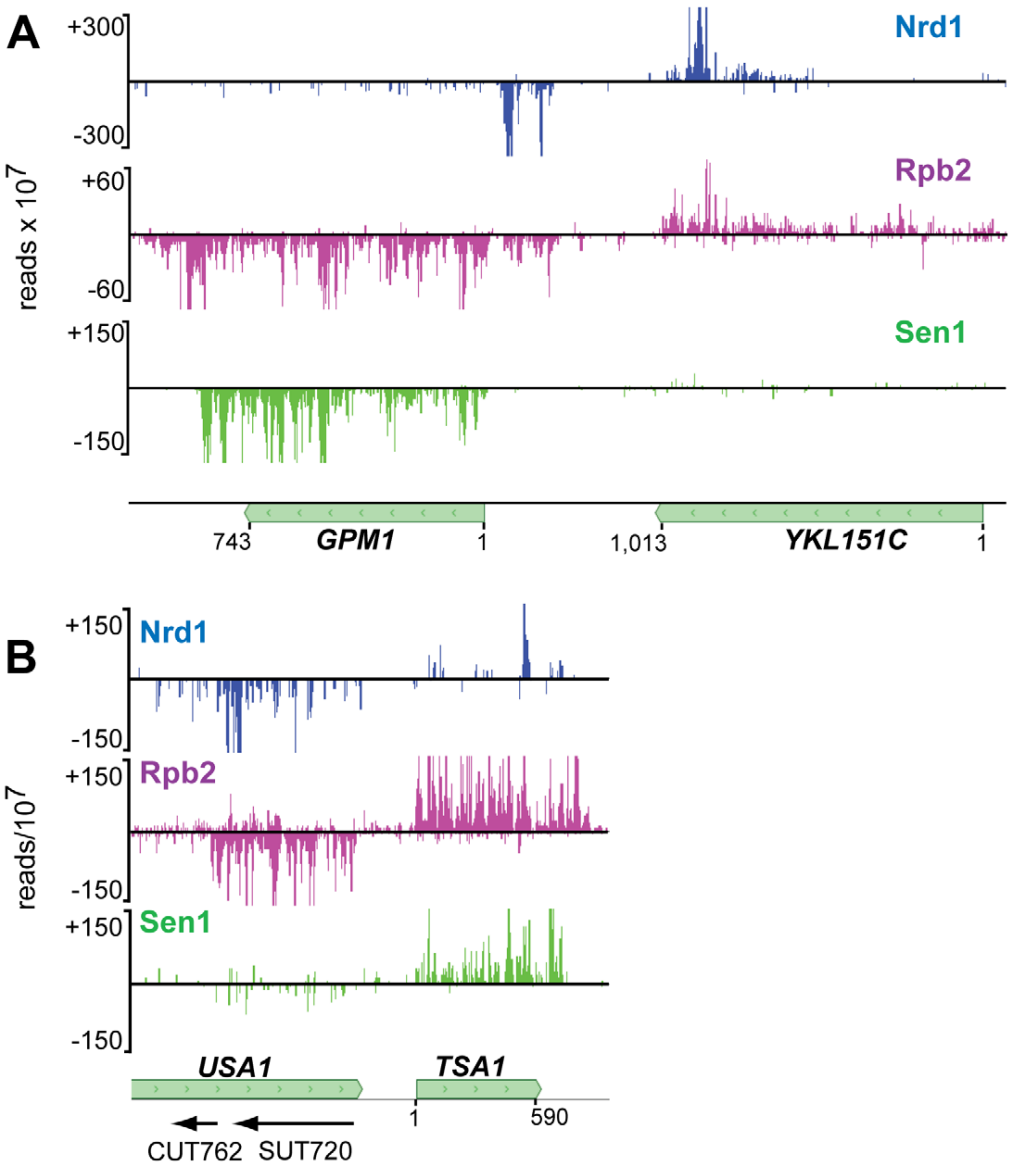
Antisense transcripts on genes that are poorly expressed. In each panel the downstream gene expresses an antisense transcript cross-linked to Nrd1 (blue) and Rpb2 (purple). Sen1 (green) cross-links poorly to the downstream antisense transcripts but cross-links to the upstream sense transcripts. In each panel the sense reads are above the midline and negative strand reads are below as indicated by the negative reads per 10^7^ on the Y axis.

In addition to sense-strand binding, we observe a large number of Nrd1 reads derived from RNAs that are anti-sense to annotated genes. Figure 4 shows that these reads are concentrated at the 3′ end of genes that are not heavily transcribed in the sense direction. Figure 6 shows Nrd1 and Rpb2 binding to antisense transcripts at the 3′ ends of the *YKL151C* and *USA1* genes. We do not observe abundant Sen1 cross-linking to the antisense transcript despite the fact that it cross-links efficiently to the sense transcript of the downstream gene. In Figure S5 we show that *YKL151C* encodes a 3′ antisense CUT. We think it is quite likely that these antisense transcripts originate from divergent transcription from the downstream promoters [32], [33] and may be involved in suppressing transcription in the sense direction of *YKL151C* and *USA1*.

### Sen1 binds to the 3′ end of mRNA transcripts

**Figure 7:**
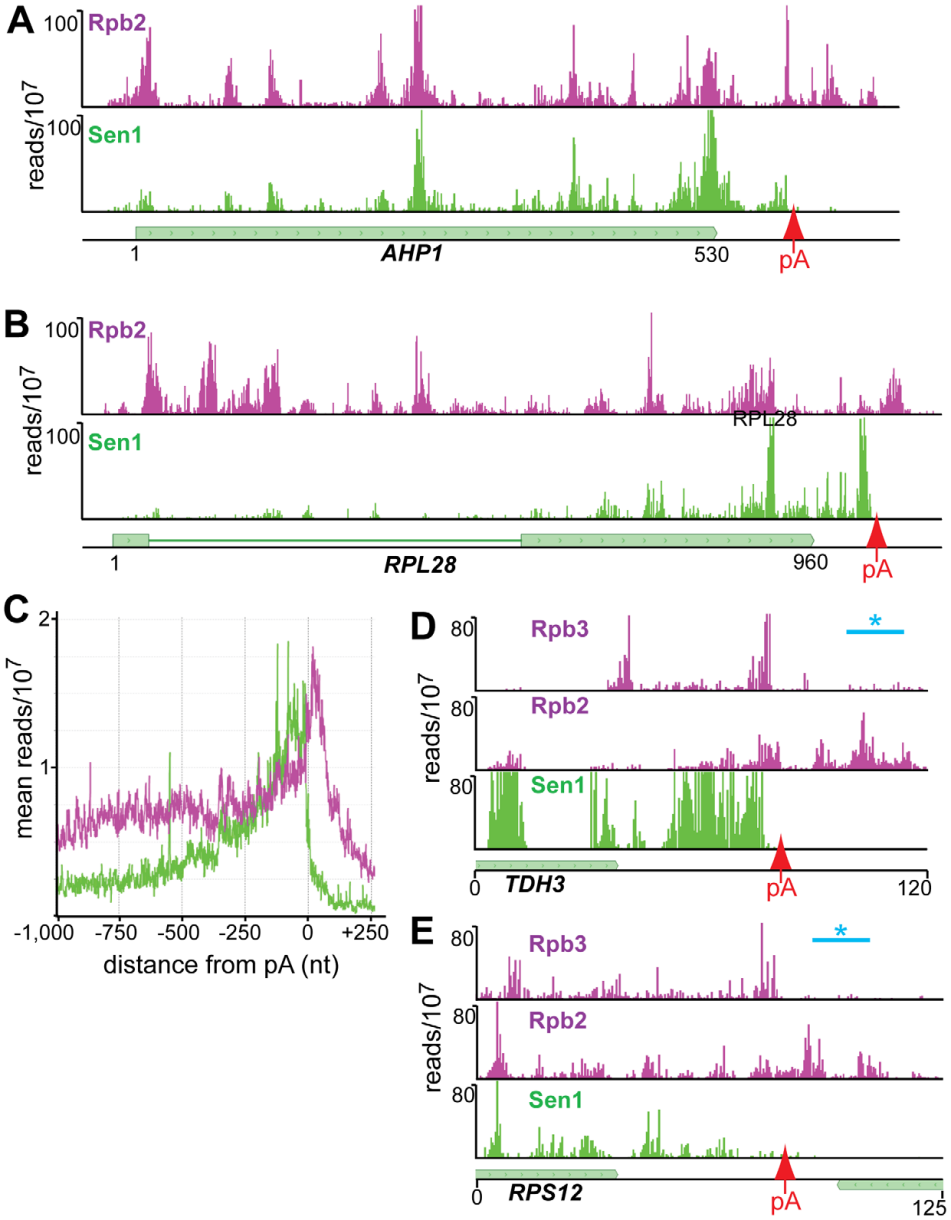
Distribution of Rpb2 and Sen1 on highly expressed genes. (A–D) Rpb2 and Sen1 reads distributed on transcripts from the *RPL28*, *RPS13*, *RPL30* and *PMA1* genes. The position of polyadenylation sites [39] is indicated by a red arrow. (E) Distribution of reads averaged over the top quartile of genes ranked by expression level. The X axis values are determined relative to the polyadenylation site [39]. Rpb2 reads are plotted in purple and Sen1 reads in green.

Our cross-linking experiments show that Sen1 cross-links to abundantly transcribed mRNAs. In Figure 7E we show examples of Sen1 binding to transcripts derived from the heavily transcribed *RPL28*, *RPS13*, *RPL30* and *PMA1* genes. Several arguments suggest that this Sen1 cross-linking occurs on nascent transcripts. First, Sen1 and Rpb2 both cross-link in clusters that are spaced along the transcript in coincident peaks. A similar clustering of Pol II-associated RNA has recently been attributed to pausing of Pol II as nucleosomes are removed from the path of the elongating polymerase [38]. Such clustering would not be expected on mature transcripts. Second, although upstream cross-links are less abundant on ribosomal gene transcripts, there is some cross-linking to intron sequences that are enriched on nascent transcripts. Taken together, these results suggest preferential binding to nascent RNA but we cannot rule out binding to unprocessed precursors that have been terminated and released from the template.

**Figure 8:**
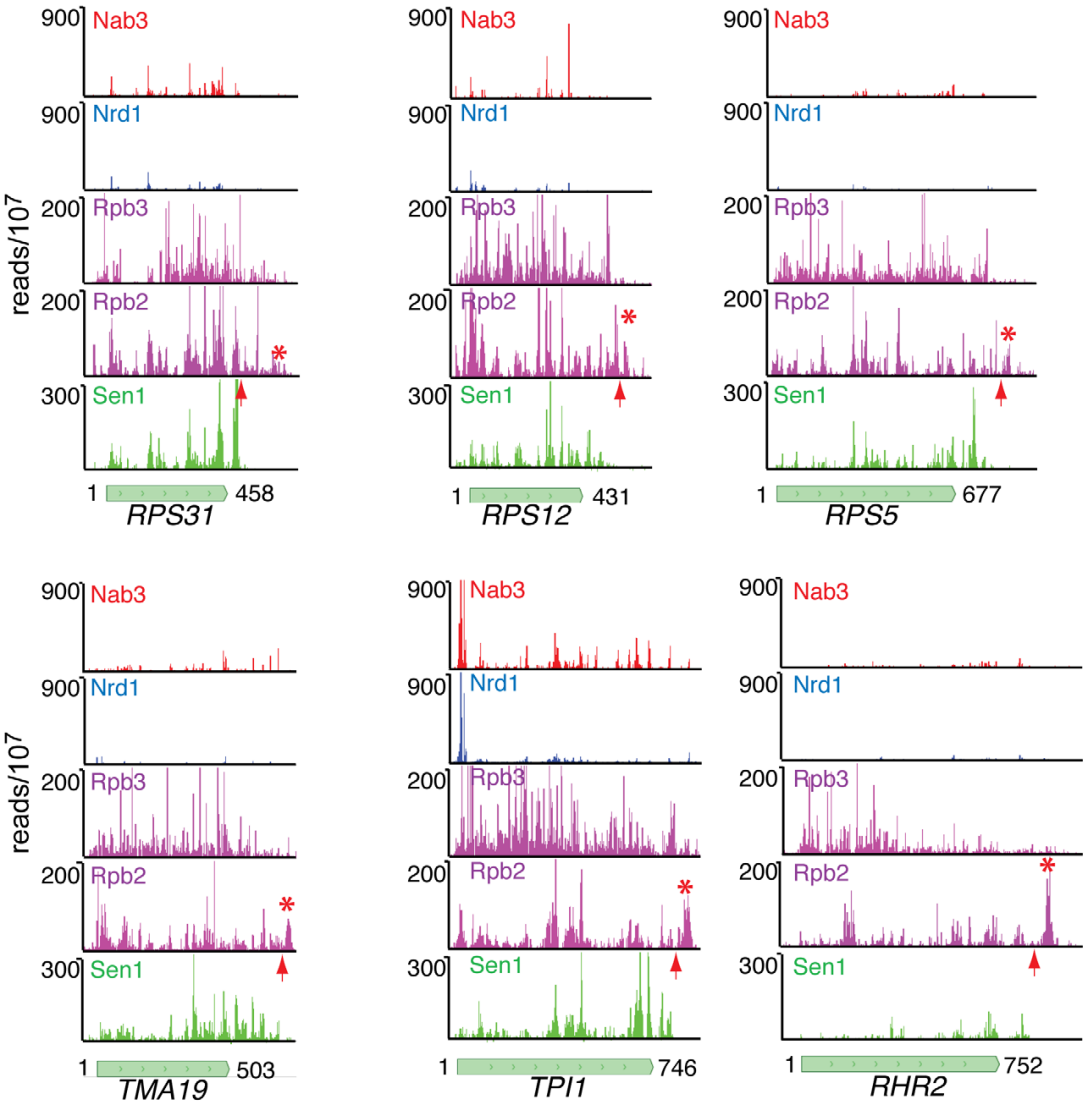
Nab3, Nrd1, Rpb2, Rpb3, and Sen1 reads distributed on a variety of genes. The missing peak of Pol II downstream of the pA sites in the NET-seq Rpb3 data [38] is indicated by an asterisk and the position of the pA site by an arrow.

We note that Sen1 peaks are stronger toward the 3′ end, especially on the ribosomal protein transcripts. This is confirmed in Figure 7D by plotting all Sen1 and Rpb2 reads with respect to the 3′ end of transcripts derived from the most heavily transcribed genes [39]. Interestingly, the peak of Sen1 is broadly distributed over the 75 nt before the polyadenylation site (pA) with few if any reads extending beyond this cleavage site. In Figure 8 we show that this downstream Sen1 cross-linking peak does not correspond to Nrd1 or Nab3 cross-linking sites. A distinct peak of Pol II is observed between 25 and 50 nt downstream of the pA site. Clearly, Pol II continues to transcribe beyond the pA site but we see little evidence for Pol II more than a few hundred bases further downstream (Figure 7E).

In Figure 8 we have also compared our Rpb2 cross-linking data set with the Net-seq data set derived from RNA non-covalently associated with affinity purified Pol II [38]. While we see a very similar pattern of reads within coding regions, we notice a difference in the pattern of Pol II downstream of the pA site. The downstream peak of Pol II that we observe in our PAR-CLIP data is often missing in the Net-seq data (Figure 8).

## Discussion

Nrd1, Nab3 and Sen1 have been shown to form a complex with Pol II [3] and to direct the termination and subsequent processing of nascent transcripts [16], [40]. We have used an in vivo cross-linking approach [18] to map the positions of these yeast RNA-binding proteins in living cells. Our protocol maps Nrd1 and Nab3 binding sites to downstream snoRNA sequences that have previously been shown to direct termination in vivo [7], [8], [10], [16], [41] thus validating our procedure. These downstream sequences are rapidly processed by the nuclear exosome [42], [43] indicating that cross-linking of Nrd1 and Nab3 occurs preferentially on nascent transcripts.

The observation that Nrd1 ChIPs to chromatin at highly expressed genes but only cross-links to a sub-set of these transcripts (Figure 1) is consistent with a model in which Nrd1, Nab3, and Sen1 enter the early Pol II elongation complex by association with the Ser5 phosphorylated CTD [4], [6]. This association places Nrd1 and Nab3 in position to monitor the nascent transcript for suitable RNA binding sites. The presence of Spt5 in the Nrd1-Nab3-Sen1 complex [3] suggests a possible role for Nrd1 and Nab3 in an early transcription elongation checkpoint that controls the transition between early inefficient elongation and the processive elongation that characterizes many Pol II genes [1], [2]. Nrd1 and Nab3 binding to the nascent transcript could help slow elongation until all components of the elongation complex are assembled.

This early elongation checkpoint may serve as a regulatory step and several yeast genes including *NRD1*, *IMD2*, *URA2*, and *SER3* contain Nrd1-bound RNAs derived from early elongation complexes. We provide evidence here that several additional genes can be added to this list including *PCF11* and *RPB10* (Figure 5); *HPT1*, *GUA1* and *ADE17* (Figure S4); and *DIP5*, *LSR1*, *RSF1*, *SWI5*, and *MEP1* (not shown). Data about the transcription start site(s) of these genes is limited leaving open the question of whether these genes are regulated by premature termination [11], alternative start site selection [12], [13], [15], or promoter occlusion by an upstream ncRNA [44].

### Nrd1 antisense binding

Another unexpected observation is that many poorly expressed protein-coding genes express Nrd1-bound 3′ antisense sequences. Previous studies in yeast have identified regulatory antisense transcripts for *IME4*, *PHO5*, *PHO84* and the Ty1 retrotransposon [45]–[49]. In the case of *PHO84* and Ty1, antisense transcripts appear to act in trans [47], [49], although in the case of *PHO84* cis-suppression is also observed [48]. In Figure 6 we show several genes with Nrd1 cross-linked anti-sense RNA peaks localized to the 3′ coding region. In each case the gene exhibiting 3′ antisense transcripts is poorly transcribed in the sense direction as determined by RNA sequencing [39] and the downstream gene is highly expressed in glucose-containing media [50]. These antisense transcripts likely result from bi-directional transcription from the downstream promoter [32], [33]. The Rpd3s histone deacetylase complex has been shown to repress antisense initiation at many promoters [38] and it is possible that the Nrd1-Nab3 non-poly(A) termination pathway prevents elongation of those antisense transcripts that lack or escape Rpd3s control. The question of whether these antisense transcripts are regulatory remains to be answered, but we note that each of these Nrd1 antisense peaks correlates with Rpb2 cross-linking sites but does not show efficient Sen1 binding. We propose that Nrd1 pauses Pol II at these sites, preventing sense strand transcription either through transcription interference or by establishment of chromatin marks that are inappropriate for transcription of the sense strand.

### Sen1 binding

The distribution of Sen1 is surprising on two counts. First, we failed to observe efficient cross-linking on some transcripts that had previously been shown to depend on Sen1 function for proper termination. *SNR13* transcripts normally terminate just downstream of the Nrd1 binding site but in a *sen1* mutant Pol II reads through into the downstream *TRS31* gene [16] and Sen1 ChIPs to this downstream region [31]. Similarly, Nrd1 autoregulation is disrupted in a *sen1* mutant with steady-state levels of Nrd1 mRNA increasing about 10-fold [11], [16]. The failure of Sen1 to efficiently crosslink to these transcripts can be explained in several ways. First, the RNA at these sites may interact with Sen1 in a manner that prevents close apposition of Sen1 amino acid side chains with 4SU residues in the bound RNA. A second possibility is that Sen1 may play a structural role, stabilizing the Nrd1-Nab3-Sen1 complex in a manner that does not require the helicase activity. In this model the *sen1* mutation may alter the structure of the complex leading to disruption of Nrd1 and/or Nab3 termination function. A third possibility is that Sen1 may be required for expression of another factor that is required for termination of non-poly(A) transcripts. Finally, the low amount of Sen1 cross-linking may indicate that low levels of Sen1 are sufficient for proper termination at some genes. Future experiments must be directed at understanding the role of Sen1 in termination of snoRNA and attenuated Pol II transcipts.

A second unexpected result from the Sen1 cross-linking data set is the widespread distribution of Sen1 on mRNA transcripts. A role for Sen1 in mRNA 3′ end formation has been suggested by previous experiments. Sen1 interacts with Glc7, a protein phosphatase that is part of the CPF complex [51], [52]. In addition, *sen1* mutants display a weak read through phenotype on some pA terminators [53], [54], [55] but not others [30]. Although a genome-wide survey of Pol II distribution in a *sen1-E1597K* mutant did not detect widespread read through of pA terminators [31] some read through was observed and our data shows that several of those genes including *RPL43B*, *RPS28A*, *RPL36B*, and *SOD1* display prominent Sen1 binding near their pA site (not shown).

Our data shows that Sen1 cross-linking occurs all along mRNA transcripts, peaking at the 3′ end (Figure 7). Sen1 interacts with the Pol II CTD [5] but whether this interaction is direct is not known. Based on the increased cross-linking toward the 3′ end of genes it is possible that Sen1 interacts with the Ser2 phosphorylated form of Pol II or another protein that binds to this phosphorylated form of the CTD.

The failure of Sen1 to cross-link downstream of the pA site would seem to rule out a rho-like termination model in which Sen1 translocates along the transcript facilitating termination upon reaching the paused downstream Pol II. Recently Mischo et al. [54] have shown that Sen1 helicase activity is required to remove R loops that form at the 3′ end of some transcripts. This proposal is based in part on the identification of DNA∶RNA hybrids downstream of the pA site [54]. Sen1 helicase activity could act to remove RNA from the DNA∶RNA hybrid and expose RNA downstream of the pA site for degradation by the 5′-3′ exonuclease Rat1. Our data argue, however, that Sen1 acts upstream but not downstream of the pA site. We can clearly observe cross-linked Pol II downstream of the pA site but there is no corresponding Sen1 cross-linking in this region. Sen1 could act upstream of the cleavage site to remove RNA from R loops and allow access of the cleavage/polyadenylation machinery and subsequently the 5′-3′ exonuclease Rat1. This model is consistent with previous experiments showing a synergistic effect of *sen1* and *rat1* mutations [53], [55].

Our data also suggest that the downstream peak of Pol II represents the Pol II termination complex. Kinetic modeling of yeast Pol II transcription suggests a termination time of about one minute [56]. This is greater than the amount of time needed to transcribe many yeast genes, suggesting that termination is the rate-limiting step for formation of some mRNAs. We suggest that the peak of Rpb2 cross-linking we observe downstream of the pA site of heavily transcribed genes (Figure 7) represents this rate-limiting Pol II elongation complex that is in the act of terminating. We note that this downstream peak of Pol II is not as prominent in the NET-seq data (Figure 8; [38]). The NET-seq data is obtained by affinity purifying Pol II elongation complexes from chromatin after digestion with DNase I. We propose that the downstream termination complex is sensitive to DNase I digestion because of an allosteric change that takes place as the elongation complex passes through the pA site [57]. Thus, the RNA is lost from these complexes and is under-represented in the NET-seq data.

## Materials and Methods

### Yeast strains

The genomic *NRD1*, *NAB3*, *SEN1* and *RPB2* genes in BY4733 were tagged with both 6His and biotin tags (HTB) [19]. The resulting strains expressed proteins with a slightly higher molecular weight that could be enriched on streptavidin beads (Figure S6). These strains displayed no abnormal growth phenotypes. A TAP-tagged *NRD1* yeast strain was used for chromatin immunoprecipitation.

### Cell growth and UV 365 nm cross-linking

For the first two data sets, yeast cells expressing HTB tagged Nrd1 or Nab3 were grown at 30°C in 2 L of synthetic complete (SC-URA) medium supplemented with 2% dextrose, 120 µM Uracil, 0.01 µM Biotin from OD_600_∼0.1 to mid-exponential phase (OD_600_∼0.5). 4SU was added to a final concentration of 300 µM and growth continued at 30°C until OD_600_∼1.5. Addition of 4SU had no effect on the growth rate of yeast during the time course of the experiment. Cells were harvested by centrifugation and the cell pellet was re-suspended in 60 ml of ice-cold water, separated into two 30 ml aliquots and placed in 145×20 mm sterile tissue culture dishes kept on ice. Cells were irradiated on ice with 365 nm UV light (0.15 J/cm^2^) in a Stratalinker 2400 (Stratagene), three times for 10 minutes each with shaking between irradiations. Cells were pooled, centrifuged, and the cell pellet was re-suspended in 5 ml of buffer-1 (8 M urea, 300 mM NaCl, 0.5% Nonidet P-40, 50 mM sodium phosphate, 50 mM Tris-HCl, pH 8.0, and EDTA-free protease inhibitor mix for His-Tag sequences (RPI)) then frozen in droplets in liquid nitrogen. Cell droplets were kept in −80°C until processed as described below.

In the analysis of the initial Nrd1 and Nab3 data sets we observed binding to a number of RNAs derived from stress-induced genes. The RNA in these early experiments was obtained from cells that were irradiated after centrifugation and re-suspension in ice-cold water [21], a procedure that is likely to induce a stress response. To eliminate this possibility we developed a second technique to irradiate cells growing in liquid media at 30°C. Two liters of growing cells were placed in a two-liter beaker on a magnetic stirrer and irradiated from a distance of 10 cm for 10 min with a UV Power–Shot 1100 Lamp. This lamp delivers 1 W/cm^2^ primarily in the in the 300–400 nM range with a peak at 365 nm. To eliminate shorter wavelength light the beaker was covered with a Pyrex baking dish. Cells irradiated in this manner were processed as described below.

### Protein–RNA purification

Protein purification was based on a previously published protocol [58]. Cell droplets were lysed in liquid nitrogen using a Spex SamplePrep 6870 freezer mill with 10 cycles of one minute of breakage and two minutes of cooling, at a frequency setting of 15 cps. Lysates were thawed at room temperature, resuspended in 5 ml of buffer-1 then sonicated using a 1/8″ microprobe tip of Branson sonifer cell disruptor Model 250/450. Sonication was performed three times at 50% power for 5 seconds with 30 second intervals at room temperature. Cell lysates were cleared by centrifugation at 40,000 rpm in Beckman L-80 ultracentrifuge at room temperature for 30 minutes using Ti 70.1 rotor. Cleared lysates were incubated with Ni-NTA agarose (QIAGEN, 500 µl slurry pre-equilibrated in buffer-1) for 3 hours at room temperature. Ni-NTA agarose was then washed in 5 ml of buffer-1; 5 ml of buffer-1, pH 6.3; and 5 ml of buffer-1, pH 6.3, +10 mM imidazole. Proteins were eluted in 8 ml of buffer-2 (8 M urea, 200 mM NaCl, 2% SDS, 50 mM sodium phosphate, 10 mM EDTA, 100 mM Tris-HCl, pH 4.3, and EDTA-free protease inhibitor mix for His-Tag sequences (RPI)). The pH of the eluate was neutralized and loaded onto streptavidin magnetic beads (New England Biolabs). A 200 µl slurry of beads was pre-equilibrated in buffer-3 (8 M urea, 200 mM NaCl, 0.2% SDS, 100 mM Tris-HCl, pH 8.0, and EDTA-free protease inhibitor mix for His-Tag sequences). After incubation overnight at room temperature the streptavidin magnetic beads were washed in 3×500 µl of buffer-3, 3×500 µl of buffer-3 with 2% SDS, 3×500 µl of buffer-3 without SDS, and then 3×500 µl of T1 ribonuclease buffer (150 mM KCl, 2 mM EDTA, 0.5 mM DTT, 50 mM Tris-HCl, pH 7.4, and EDTA-free protease inhibitor mix for His-Tag sequences (RPI Crop.)). The streptavidin magnetic beads were resuspended in 0.5 ml of T1 buffer before RNase T1 (Fermentas) was added to obtain a final concentration of 40 U/ml and the bead suspension was incubated at room temperature for 15 minutes. Beads were washed three times with 500 µl of T1 wash buffer (500 mM KCl, 0.05% NP40, 0.5 mM DTT, 50 mM Tris-HCl, pH 7.8, and EDTA-free protease inhibitor mix for His-Tag sequences (RPI Corp.)) and three times with 500 µl of polynucleotide kinase (PNK) buffer (50 mM NaCl, 10 mM MgCl_2_, 5 mM DTT, 50 mM Tris-HCl, pH 7.4, and EDTA-free protease inhibitor mix for His-Tag sequences). Beads were resuspended in 160 µl of PNK buffer before Thermosensitive Alkaline Phosphate (TSAP) (Promega) was added to obtain a final concentration of 0.15 U/µl, and SuperRNase inhibitor (Ambion) was added to obtain a final concentration of 1 U/µl. The bead suspension was incubated for 30 minutes at 37°C then was washed once with 500 µl of buffer-3 without SDS, and 3 times with 500 µl of PNK buffer.

### cDNA library construction from crosslinked RNA

Streptavidin magnetic beads from the previous step were resuspended in 200 µl of PNK buffer then γ-^32^P-ATP (MP Biomedicals) was added to obtain a final concentration of 0.5 µCi/µl and T4 PNK (New England Biolabs) was added to obtain a final concentration of 1 U/µl. The bead suspension was incubated at 37°C for 30 minutes before non-radioactive ATP was added to obtain a final concentration of 100 µM, the incubation was continued for 10 minutes at 37°C. The bead suspension was washed 4 times with 650 µl of T4 RNA Ligase2 truncated (Rnl2 (1–249)) buffer (2 mM MgCl_2_, 1 mM DTT, 50 mM Tris-HCl, pH 7.5) (New England Biolabs).

Streptavidin magnetic beads from the previous step were resuspended in 19 µl of Rnl2 (1–249) buffer combined with 19 µl of 50% Polyethylene Glycol 8000 (PEG 8000) (Promega). To the bead suspension was added 2 µl of 100 µM adenylated 3′ adapter oligodeoxynucleotide (AppATCTCGTATGCCGTCTTCTGCTTGTC; IDT), 0.4 µl of 1 M MgCl_2_, 2.5 µl of Rnl2 (1–249) (200 U/µl) (New England Biolabs), 1.25 µl of RNase inhibitor (40 U/µl) (Invitrogen). The bead suspension was incubated at room temperature for 4 hours then washed once with 500 µl of buffer-3 without SDS, and 3 times with 500 µl of PNK buffer. The washed streptavidin magnetic beads were re-suspended in 38 µl of PNK buffer, then to the bead suspension was added 2 µl 100 µM 5′ adapter oligonucleotide (GUUCAGAGUUCUACAGUCCGACGAUC), 1.25 µl of RNase inhibitor (40 U/µl), 2.5 µl of T4 RNA ligase (10 U/µl) (Fermentas), 3 µl of 10 mM ATP (New England Biolabs). The bead suspension was incubated overnight at 16°C then washed once with 500 µl of buffer-3 without SDS, and 3 times with 500 µl of Protease K buffer (75 mM NaCl, 6.25 mM EDTA, 1% SDS, 50 mM Tris-HCl, pH 7.5). The streptavidin magnetic beads were resuspended in 200 µl of protease K buffer follow by the addition of Protease K (Ambion) to the final concentration of 1.2 mg/ml. After incubation at 55°C for 30 minutes, the supernatant was transferred to a new tube and another 200 µl of protease K buffer was added to the streptavidin magnetic beads. The incubation was continued for 5 minutes at 55°C before the supernatant was combined and the RNA was recovered by acidic phenol/chloroform extraction followed by a chloroform extraction and an ethanol precipitation. The pellet was dried and then dissolved in 12 µl of DPEC water. The recovered RNA was divided into 3×4 µl aliquots and kept in −80°C until further preparation.

The recovered RNA was used to synthesize a cDNA library. Time course PCR amplification was performed in order to determine the optimum number of cycles for amplifying the cDNA library. One aliquot of recovered RNA was taken out of −80°C to thaw on ice, then reverse transcription oligonucleotide (CAAGCAGAAGACGGCATACGA) was added to the final concentration of 5 µM. The mixture was briefly centrifuged, heated at 70°C on a preset thermal cycler for 2 minutes and the tube was then placed on ice. To the iced tube containing the primer-annealed template RNA was added 4 µl of the premixed reverse transcription reaction mixture containing 2 µl of 5 X first strand buffer (375 mM KCl, 15 mM MgCl_2_, 250 mM Tris-HCL, pH 8.3) (Invitrogen), 0.5 µl of 12.5 mM dNTP mix (Fermentas), 1 µl of 0.1 M DTT (Invitrogen), and 0.5 µl of RNase inhibitor (40 U/µl) (Invitrogen). The tube was heated on the preset thermal cycler at 48°C for 3 minutes then Superscript II reverse transcriptase (Invitrogen) was added to the final concentration of 20 U/µl, the incubation was continued for 1 hour on the preset thermal cycler at 44°C. Time course PCR was carried out on a 50 µl scale using cDNA from the previous step and Phusion DNA polymerase (Finnzymes) (10 µl of the cDNA, 0.5 µl of 25 mM dNTP Mix, 10 µl of 5 x Phusion HF buffer (Finnzymes), 1 µl of 100 µM primers, 0.5 µl of 2 U/µl Phusion DNA polymerase). PCR cycle conditions of 10 s at 98°C, 30 s at 60°C, 15 s at 72°C were used. Aliquots of 6 µl were removed every other cycle starting with cycle number 14 by temporarily pausing the PCR cycle at the end of the 72°C step. The maximum cycle of the PCR was set at 30 cycles. PCR aliquots were analyzed on a 6% Novex TBE PAGE gel (Invitrogen) and the optimal cycle number for cDNA amplification was chosen as five cycles prior to reaching the saturation level of PCR amplification. The optimal cycle number PCR was performed on a 50 µl scale using cDNA prepared from another aliquot of recovered RNA (4 µl). The amplified cDNA product was separated on a 6% Novex TBE PAGE gel (Invitrogen) and the band of interest was excised and eluted using 1 x gel elution buffer (Illumina). RNA fragments with both adapters produce PCR fragments that span a range of 100–150 nt. The 5′-adapter-3′-adapter products without inserts may be detected after amplification of the cDNA as an additional PCR band at around 75 nt in length. The eluted DNA from gel extraction was ethanol precipitated followed by DNA analysis using Agilent 2100 Bioanalyzer. DNA was sequenced using an Illumina GAII sequencer (University of California, Riverside).

### Bioinformatic analysis

Sequences were trimmed using the function trimLRPatterns from the ShortRead package in Bioconductor [59]. Reads were aligned to the *Sacchromyces cerevisiae* genome using the short read aligner Bowtie [60]. The bowtie alignment allowed the read to have up to 1 mismatch and align once to the genome. Reads that aligned more than once to the genome will be discussed elsewhere [22]. All figures were made using the Mochiview genome browser [29]. The processed wig files for each dataset, along with fasta file of the yeast genome for alignment, can be downloaded from Gene Expression Omnibus [61] under the series number GSE31764 (http://www.ncbi.nlm.nih.gov/geo/query/acc.cgi?acc=GSE31764).

### Chromatin immunoprecipitation

Cells containing a TAP-tagged *NRD1* gene were grown in yeast extract peptone dextrose (YPD) to an absorbance of about 0.8 and cross-linked with formaldehyde for 20 min at room temperature and processed for chromatin immunoprecipitation according to standard protocols [62]. ChIP and Input DNA were first amplified using linker adapted random primer and sequenase 2 and further amplified and labeled using PCR and Cy3- and Cy5- CTP, respectively. Cy dye labeled ChIP and Input DNA was combined with Agilent aCGH/ChIP hybridization buffer containing 2x Hi-RPM Hyb Buffer, Agilent blocking agent and Human cot-1 DNA and hybridized to Agilent 244K yeast tiling array (G4491A) for 40 hours on MAUI hybridization system with constant mixing. The hybridized array was washed using Agilent aCGH/ChIP-on-chip array washing buffer kit (5188–5266) and scanned on Axon GenePix Scanner (GenePix A4300) and the raw data extracted using GenePix Pro 6.0. Preliminary analysis of the ChIP data was carried out using CisGenome to identify ChIP enriched regions.

### Yeast RNA analysis

Total RNA was extracted from yeast with hot acid phenol and run on a 1% denaturing formaldehyde MOPS agarose gel, visualized by ethidium bromide and Northern blotted as previously described [11]. Samples with clear rRNA bands and no visible degradation were analyzed by quantitative real-time RT-PCR. RNA was treated with turbo-DNA-free (Ambion) according to the manufacturer's instructions for the most stringent treatment. Reverse transcription was performed using the iScript cDNA Synthesis Kit (BioRad). Real-time PCR was performed in triplicate 20 µl reactions on a CFX96 Real-time PCR detection system (BioRad) using iQ SYBR green supermix (BioRad) according to the manufacturer's instructions. Data from at least two replicate experiments were pooled using the Gene Study feature of the CFX96 real-time software, which normalizes for fluorescence intensity differences between plates. Expression was normalized to both *ACT1* and 18S ribosomal RNA and the ratios were graphed relative to the wild-type control sample, which was set to 1 for each gene. Error bars represent the positive and negative range of the standard error of the mean.

### Treatment of tet-promoter strains

Tet-promoter yeast strains (Open Biosystems) and a control strain lacking a tet-responsive promoter were seeded to an initial *A*_600_ of 0.06 in YEPD. Cultures were grown at 30° for 2.5 hrs prior to the addition of doxycycline at a final concentration of 10 µg/ml and were grown an additional five hrs before collection.

## Supporting Information

Figure S1 Distribution of Nrd1 and Nab3 cross-links on snR13 and Nrd1 transcripts. (A) Nab3 (red) and Nrd1 (blue) reads are aligned to the region downstream of the *SNR13* gene. The sequence corresponding to the main peak is shown below the plot with an arrow indicating the most frequently cross-linked base. Nrd1 and Nab3 binding motifs identified in our earlier genetic selection are indicated above the transcript and the arrow indicates the most frequent T to C transition. (B) Nrd1 and Nab3 reads aligned to the Nrd1 gene. This binding region correlates with earlier work showing that sequences upstream of and including the 5′ part of the coding region are necessary for autoregulation of Nrd1 expression.
(EPS)

Figure S2 Cross-linking of Nrd1, Rpb2 and Sen1 to the *IMD2* gene. The transcription start site is indicated by an arrow on the gene map.
(EPS)

Figure S3 Cross-linking of Sen1, Rpb2, Nab3 and Nrd1 to selected CUTs and SUTs. (A) Cross-linking to NEL025c, the original CUT (SUT?) [34] (B and C) Cross-linking to CUTs described in [9]. Note that the antisense CUT in the 3′ end of *FMP40* is not annotated in the Steinmetz or Jacquier databases [31], [32]. (D and E) Cross-linking to some CUTs but not others. In each of these figures there is one CUT cross-linked while another is not. This is also observed in A and B. (F) Highly expressed CUT585 cross-links efficiently to Nrd1 and Nab3 but not Sen1.
(EPS)

Figure S4 Binding of Nrd1 to the upstream regions of genes involved in nucletide metabolism. (A) *GUA1* encoding GMP synthase. (B) *ADE17* encoding both 5-aminoimidazole-4-carboxamide ribonucleotide transformylase and inosine monophosphate cyclohydrolase activities. (C) *HPT1* encoding hypoxanthine-guanine phosphoribosyltransferase.
(EPS)

Figure S5 NRD1-dependent termination of a transcript antisense to YKL151C. RNAs produced in strains with wild-type *NRD1* (*NRD1HA*) or mutant *nrd1* (*nrd1-102HA*) with and without the deletion of the exosome gene *RRP47* were probed with a radio-labeled T7 transcript complimentary to the antisense RNA. *SCR1* is probed as a loading control.
(EPS)

Figure S6 Western blot of proteins derived from tagged Nrd1, Nab3, Rpb2 and Sen1 strains. (A) Protein extracts were run on SDS-PAGE and transferred to nitrocellulose. Nrd1 (green) was detected with a rabbit polyclonal antibody and Nab3 (red) with a mouse monoclonal antibody. Secondary antibodies were labeled with IR dyes and detected with a LiCor Odyssey scanner. (B) Proteins in cell extracts were affinity purified on streptavidin beads, eluted in SDS sample buffer, run on SDS PAGE, blotted and probed with an anti-His-tag antibody anti-RGS(H)_4_, (Qiagen). The asterisk indicates the position of the Rpb2 tagged protein. Due to its low abundance or instability we were unable to observe Sen1-HTB in extracts by Western Blot. (C) Silver stain of Sen1-HTB purified on strepavidin beads.
(EPS)

Table S1 Distribution of sequence reads corresponding to different classes of RNAs. The distribution of reads from five different data sets is presented as both the number of reads and the percentage of total reads. The data discussed in this publication have been deposited in NCBI's Gene Expression Omnibus [61] and are accessible through GEO Series accession number GSE31764 (http://www.ncbi.nlm.nih.gov/geo/query/acc.cgi?acc=GSE31764).
(DOC)

Table S2 The top 100 Nab3 cross-linked sites.
(XLSX)

Table S3 The top 100 Nrd1 cross-linked sites.
(XLSX)
