# Supplementary material for: Transcriptome-Wide Binding Sites for Components of the Saccharomyces cerevisiae Non-Poly(A) Termination Pathway: Nrd1, Nab3, and Sen1
Source: PLoS Genet. 2011 Oct 20;7(10):e1002329. doi: 10.1371/journal.pgen.1002329 (PMC3197677; doi:10.1371/journal.pgen.1002329)
Supplement: Table S1 — Distribution of sequence reads corresponding to different classes of RNAs. The distribution of reads from five different data sets is presented as both the number of reads and the percentage of total reads. The data discussed in this publication have been deposited in NCBI's Gene Expression Omnibus [61] and are accessible through GEO Series accession number GSE31764 (http://www.ncbi.nlm.nih.gov/geo/query/acc.cgi?acc=GSE31764). (DOC) [file pgen.1002329.s007.doc]

Supplemental Table 1

| Sample Name – Crosslinker Used | Total # of reads sequenced | Processed reads with 3’ adapter trimmed  (% of total reads) | Reads that  uniquely aligned  (% of processed reads) | Reads aligned to rDNA  (% of processed reads) | Reads aligned to tRNA  (% of processed reads) |
| --- | --- | --- | --- | --- | --- |
| Nrd1 – Stratalinker | 30,487,678 | 7,204,155 (24%) | 2,574,062 (36%) | 1,272,222  (18%) | 757,333  (11%) |
| Nab3 – Stratalinker | 28,060,633 | 9,428,610 (34%) | 3,930,441 (42%) | 1,823,479  (19%) | 638,370  (7%) |
| Nrd1 – Arc Lamp | 20,815,110 | 8,670,990 (42%) | 2,522,325 (29%) | 817,733  (9%) | 66,262  (1%) |
| Sen1-Arc Lamp | 41,901,726 | 9,941,95  (23%) | 2,086,863  (23%) | 1,921,364  (19%) | 480,779  5% |
| Rpb2 – Arc Lamp | 40,767,741 | 11,997,448 (29%) | 5,163,785 (43.04%) | 729,313  (6%) | 86,166  (1%) |
